# OMIP‐056: Evaluation of Human Conventional T Cells, Donor‐Unrestricted T Cells, and NK Cells Including Memory Phenotype by Intracellular Cytokine Staining

**DOI:** 10.1002/cyto.a.23753

**Published:** 2019-03-28

**Authors:** One Dintwe, Shamiska Rohith, Katharine V. Schwedhelm, M. Juliana McElrath, Erica Andersen‐Nissen, Stephen C. De Rosa

**Affiliations:** ^1^ Cape Town HVTN Immunology Laboratory Cape Town South Africa; ^2^ Vaccine and Infectious Disease Division Fred Hutchinson Cancer Research Center Seattle Washington, 98109; ^3^ Department of Medicine University of Washington Seattle Washington, 98195; ^4^ Department of Laboratory Medicine University of Washington Seattle Washington, 98195

**Keywords:** cytometry, human PBMC, T cells, MAIT cells, γδ T cells, NK cells, memory, intracellular cytokines

## Abstract

A 26‐color staining panel was developed to profile human antigen‐specific T cells in an intracellular cytokine staining (ICS) assay using peptide pools to various antigens of interest. In addition to multiple functional markers, the panel includes differentiation/activation markers and markers to assess γδ, mucosal‐associated invariant T, and NK T cells as well as conventional NK cells. Panel optimization was performed using previously cryopreserved PBMC from healthy adults, and then, expression of key functional markers in the panel was cross‐validated against a validated ICS assay used in the HIV Vaccine Trials Network (HVTN). The panel is currently being used to evaluate the responses to tuberculosis and malaria vaccine candidates in volunteers from different geographic areas. © 2019 The Authors. *Cytometry Part A* published by Wiley Periodicals, Inc. on behalf of International Society for Advancement of Cytometry.

## Graphical abstract

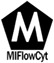

Despite extensive research and clinical testing, highly effective vaccines against complex pathogens such as HIV, tuberculosis (TB), and malaria have not yet been developed. An impediment to this development is the lack of known immune correlates of protection to the pathogens. Multicolor flow cytometry, and in particular, new technologies allowing up to 28‐color flow cytometry, could enable identification of novel immune correlates of risk or protection.

Within our HIV Vaccine Trials Network (HVTN) Laboratory Center, intracellular cytokine staining (ICS) is used to quantify and profile T‐cell responses to vaccine candidates. The laboratories adhere to good clinical laboratory practices (GCLP), and thus, our ICS assays are validated for the primary functional markers. A prerequisite of applying this new panel in vaccine studies was to retain the same sensitivity for expression of the functional biomarkers IFN‐γ, IL‐2, TNF‐α, and CD40L as in prior ICS assays widely used within the HVTN 1, 2.

Because this staining panel was designed to elucidate potential immune correlates in a TB vaccine efficacy clinical trial, markers proposed for the panel were prioritized based on the types of immune responses that could be important for control of TB (in order of priority): 1) lineage and viability: CD3, CD4, CD8, CD14, viability; 2) functional markers: IFN‐γ, IL‐2, TNF‐α, CD40L, IL‐17, granzyme A, Th2 cytokines (IL‐4/IL‐13 combined for one detector); 3) memory markers: CCR7, CD45RA; 4) additional lineage markers: γδ TCR, CD56, CD16; 5) MAIT cells: CD161, CD26, Vα7.2; 6) activation: HLA‐DR; 7) chemokine receptor for helper T cell classification: CXCR3, CCR6; 8) additional functional markers: IL‐22, perforin; and 9) differentiation markers: KLRG‐1, PD‐1.

Although the BD FACSymphony instrument currently includes capability for 28 markers, designing panels that use all available detectors can be challenging. Because we were initially concerned that there is a limited choice for conjugated reagents for the G710 detector, and the fluorochrome commonly used for this detector (PE‐Cy5.5) is spectrally similar to fluorochromes detected in the B710 detector (PerCP‐Cy5.5 and BB700), our aim was to develop a panel for 27 markers (or colors). Many panel versions were tested, and one detector (B610 for detection of BB630) was shown to cause many spreading issues into other detectors, as assessed by fluorescence minus one (FMO) testing. The B610 detector was therefore not used, although additional optimization would likely have enabled use of this detector. Thus, the final panel included 26 markers (Tables 1 and 2). Among the list of markers above, PD‐1 was considered lower priority and was dropped. Figure 1 shows an example of the staining profile for PBMC stimulated with staphylococcal enterotoxin B. Although this panel was initially developed for peptide pool antigens, other types of antigens such as recombinant proteins or whole pathogens have also be tested using this panel. Further developmental strategies and details for the panel may be found in the online material.

**Table 1:** Summary table for application of OMIP‐056

Purpose	Characterization of antigen‐specific CD4+, CD8+, γδ, MAIT, and NK T cells and NK cells
Species	Human
Cell types	Cryopreserved PBMC
Cross‐references	OMIP‐014, OMIP‐025

MAIT, mucosal‐associated invariant T cells; NK, natural killer; PBMC, peripheral blood mononuclear cells.

**Table 2:** Reagents used for OMIP‐056

Detector	Fluorochrome	Specificity	Clone	Purpose
B515	FITC	Perforin	B‐D48	Function
B660	BB660	CD14	MϕP9	Monocytes
B710	BB700	IL2	MQ1‐17H12	Function
G575	PE	IL22	22URTI	Function
G610	PE‐Dazzle 594	KLRG1 (MAFA)	SA231A2	Differentiation
G660	PE‐Cy5	CXCR3	1C6/CXCR3	T helper class
G710	PE‐Cy5.5	CD56	CMSSB	NK, NKT
G780	PE‐Cy7	CD154	24–31	Function
R660	APC	IL4	MP4‐25D2	Function
		IL13	JES10‐5A2	
R710	Alx700	Granzyme A	CB9	Function
R780	APC‐Cy7	TCRvα7.2	3C10	MAIT
U395	BUV395	CD3	UCHT1	T cell lineage
U450	UViD	Viability	NA	Viability
U500	BUV496	CD45RA	HI100	Differentiation
U570	BUV563	CD8	RPA‐T8	T cell lineage
U660	BUV661	HLA‐DR	G46‐6	Activation
U730	BUV737	IL17a	N49‐653	Function
U780	BUV805	CD4	RPA‐T4	T cell lineage
V450	V450	IFNy	B27	Function
V510	BV510	TCR γδ	11F2	γδ T cell lineage
V570	BV570	CD16	3G8	NK, NKT
V610	BV605	CCR6 (CD196)	11‐A9	T helper class
V655	BV650	CD161	DX12	MAIT
V710	BV711	CD26	M‐A261	MAIT
V750	BV750	TNFα	MAb11	Function
V780	BV785	CCR7 (CD197)	G043H7	Differentiation

APC, allophycocyanin; Alx, Alexa; BB, brilliant blue, BUV, brilliant ultraviolet; BV, brilliant violet; Cy, cyanine; FITC, fluorescein isothiocyanate; PE, R‐phycoerythrin; UViD, LIVE/DEAD fixable ultraviolet dead cell stain.

**Figure 1:**
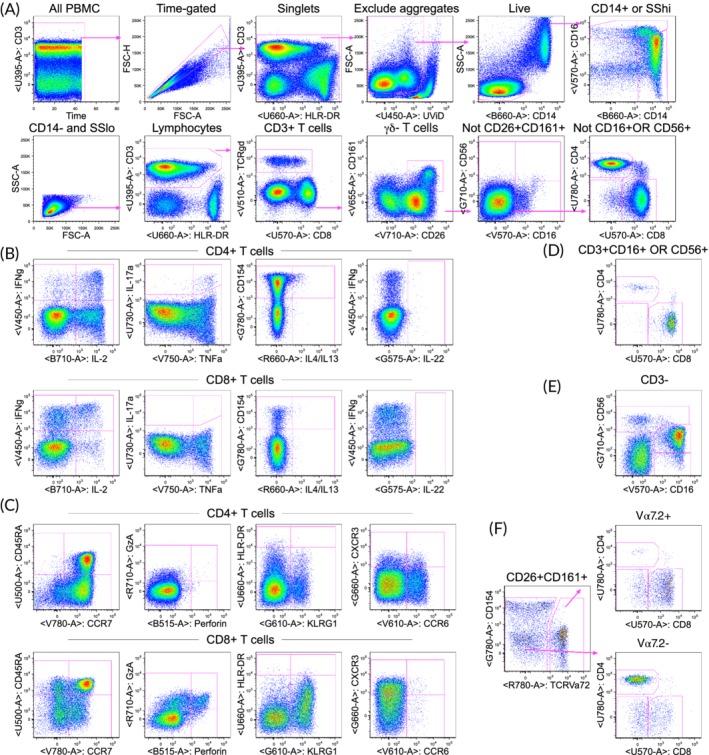
Example of the staining and gating strategy for PBMC stimulated with staphylococcal enterotoxin B (SEB). PBMC from a healthy adult were stimulated for 6 h with SEB. (A) Gating hierarchy to identify lineages. Initial gating on time (seconds) to exclude any events early in collection if there are pressure fluctuations, singlet gating on forward scatter height vs. area, exclusion of aggregates (only one example shown, but several sequential gates on various parameters are used), and live cell gating. Monocytes are gated as either CD14+ or high for side scatter and the upper right graph shows three monocyte subsets based on CD14 vs. CD16. Non‐monocytes are gated as CD14‐SS^lo^ and then scatter gated on lymphocytes. The gating scheme avoids any overlapping subsets as shown in supplemental figure 4. Thus, conventional CD4+ and CD8+ T cells are gated as CD3+, then γδ‐, not CD26 + CD161+ (containing MAIT cells), and not CD16+ OR CD56+ (containing NKT cells). (B) Functional markers for CD4+ and CD8+ T cells. A gate is applied for each cytokine, and Boolean gates are created to identify cells expressing different combinations of markers. The single function gates are sometimes chosen vs. a parameter that displays some FMO spreading to allow for angled gates. Most gates are copied, applied to all lineages, and then cloned so that any changes to the gate on one lineage changes that gate for all lineages. However, the IL‐22 gate was uniquely lower for CD4 T cells (compared to other lineages) since the CD8 reagent caused some spreading into IL‐22 and thus requiring a higher gate for all other lineages that express CD8. (C) Additional functional and non‐functional markers for CD4+ and CD8+ T cells. Perforin and granzyme A are constitutive but can be examined as co‐expression with another functional marker. (D) NK T cells gated as CD16+ OR CD56+ on CD3+ γδ‐ T cells. Expression of CD4 vs. CD8 is shown, but all the other markers are also gated on the NK T cells. (E) NK cell subsets defined by CD16 vs. CD56 on CD3‐ lymphocytes. (F) MAIT cells identified as CD3+ γδ‐ CD26 + CD161+ and then Vα7.2+. The Va7.2+ cells are predominantly CD8+; however, the Vα7.2‐ cells are predominantly CD4+ and are likely not MAIT cells. For all gates, none are placed lower than that defined by FMO controls. Some gates are placed higher to improve the specificity, for example, for the functional markers based on the background as observed in the unstimulated controls (Online Fig. 6). The labels above each graph indicate the cells included in that graph.

## similarity to published omips

This panel is unique in the combination of functional and phenotypic markers, but it can be considered an expansion of two of our prior ICS assays, OMIP‐014 1, and OMIP‐025 2.

## funding

Grant sponsors: National Institute of Allergy and Infectious Diseases funding for the HIV Vaccine Trials Network Laboratory Center UM1 AI068618 (to MJM); Bill and Melinda Gates Foundation investments OPP1066048, OPP1088952, and OPP1099507 (to MJM); University of Washington/Fred Hutch Center for AIDS Research P30 AI027757.

## Supporting information


**Appendix S1:** Supporting information
