# Supplementary material for: OMIP‐056: Evaluation of Human Conventional T Cells, Donor‐Unrestricted T Cells, and NK Cells Including Memory Phenotype by Intracellular Cytokine Staining
Source: Cytometry A. 2019 Mar 28;95(7):722–5. doi: 10.1002/cyto.a.23753 (PMC6663658; doi:10.1002/cyto.a.23753)
Supplement: Supplementary file 1 — Appendix S1: Supporting information [file CYTO-95-722-s001.docx]

**Online material OMIP-0XX:** Evaluation of human conventional T cells, donor-unrestricted T cells and NK cells including memory phenotype by intracellular cytokine staining

**Online Table 1. Instrument configuration.**

| Laser  Wavelength (nm) | Laser Power (mW) | Laser Type | Detector | Spectral Range for Detector (nm) | Dichroic LP Filter (nm) | Band Pass (nm) | Example Fluorochrome |
| --- | --- | --- | --- | --- | --- | --- | --- |
| 637 (Red) | 200 | DPSS | R780 | 750-810 | 750 | 780/60 | APC-Cy7 |
|  |  |  | R710 | 723-753 | 685 | 730/45 | Alexa700 |
|  |  |  | R660 | 655-685 | 630 | 670/30 | APC |
| 532 (Green) | 200 | DPSS | G780 | 750-810 | 750 | 780/60 | PE-Cy7 |
|  |  |  | G710 | 690-730 | 600 | 710/40 | PE-Cy5.5 |
|  |  |  | G660 | 685-735 | 690 | 710/50 | PE-Cy5 |
|  |  |  | G610 | 640-680 | 635 | 660/40 | PE-Dazzle594 |
|  |  |  | G575 | 600-620 | 600 | 610/20 | PE |
| 488 (Blue) | 200 | DPSS | B780 | 760-800 | 740 | 780/40 | BB790 |
|  |  |  | B710 | 660-760 | 690 | 710/50 | BB700 |
|  |  |  | B660 | 580-620 | 635 | 600/40 | BB660 |
|  |  |  | B610 | 600-620 | 600 | 610/20 | BB630 |
|  |  |  | B515 | 505-525 | 495 | 515/20 | FITC |
| 405 (Violet) | 200 | DPSS | V780 | 754-816 | 770 | 785/62 | BV785 |
|  |  |  | V750 | 735-765 | 735 | 750/30 | BV750 |
|  |  |  | V710 | 690-730 | 685 | 710/40 | BV711 |
|  |  |  | V655 | 650-670 | 630 | 660/20 | BV650 |
|  |  |  | V610 | 595-615 | 580 | 605/20 | BV605 |
|  |  |  | V570 | 563-588 | 550 | 575/25 | BV570 |
|  |  |  | V510 | 505-525 | 495 | 515/20 | BV510 |
|  |  |  | V450 | 563-588 | 550 | 575/25 | BV450 |
| 355 (Ultraviolet) | 60 | DPSS | U785 | 754-816 | 755 | 785/62 | BUV805 |
|  |  |  | U740 | 723-758 | 710 | 740/35 | BUV737 |
|  |  |  | U660 | 640-680 | 635 | 660/40 | BUV661 |
|  |  |  | U570 | 550-590 | 550 | 570/40 | BUV563 |
|  |  |  | U500 | 500-530 | 470 | 515/30 | BUV495 |
|  |  |  | U450 | 425-475 | 410 | 450/50 | UViD |
|  |  |  | U395 | 365-393 | None | 379/28 | BUV395 |

The OMIP-0XX panel was optimized using the FACSymphony A5 (X50) cytometer (BD Biosciences) with a five-laser configuration and the listed optical elements. For each laser, the long pass (LP) dichroic filter in each detector sends longer wavelengths through the respective band pass filter and reflects shorter wavelengths down the optical path to the next dichroic filter. DPSS, diode-pumped solid-state laser.

**Online Table 2. Commercially available reagents used in OMIP-0XX.**

| **Specificity** | **Fluorochrome** | **Ab clone** | **Vendor** | **Catalog #** | **Titer^a^** | **Staining conditions^b^** | |  |
| --- | --- | --- | --- | --- | --- | --- | --- | --- |
|  |  |  |  |  |  | **Time** | **Step** | |
| CD4 | BUV805 | RPA-T4 | BD | 564910 | 0.5 | 30 | Intracellular | |
| IFN-γ | V450 | B27 | BD Pharm | 560371 | 0.25 | 30 | Intracellular | |
| Dead cells | U450 | NA | Invitrogen | L34962 | 0.1 | 20 | Surface | |
| CD14 | BB660 | M⏀P9 | BD Horizon | 624295 | 0.1 | 25 | Surface | |
| CD3 | BUV395 | UCHT1 | BD | 563546 | 0.15 | 30 | Intracellular | |
| CD154 | PE-Cy7 | 34-31 | Biolegend | 310832 | 0.05 | 30 | Intracellular | |
| CD56 | PE-Cy5.5 | CMSSB | eBioscience | 35-0567-42 | 5 | 25 | Surface | |
| CD8 | BUV563 | RPA-T8 | BD Horizon | 565696 | 0.1 | 30 | Intracellular | |
| CCR7 | BV785 | G043H7 | Biolegend | 353230 | 2.5 | 25 | Surface | |
| TNF-α | BV750 | MAb11 | BD Horizon | 566359 | 0.05 | 30 | Intracellular | |
| IL-4 | APC | MP4-25D2 | Biolegend | 500812 | 0.5 | 30 | Intracellular | |
| IL-2 | BB700 | MQ1-17H12 | BD Horizon | 566405 | 0.25 | 30 | Intracellular | |
| Perforin | FITC | B-D48 | Biolegend | 353310 | 1 | 30 | Intracellular | |
| IL-22 | PE | 22URTI | ebioscience | 12-7229-42 | 0.5 | 30 | Intracellular | |
| CD45RA | BUV496 | HI100 | BD | 624283 | 0.05 | 25 | Surface | |
| CXCR3 | PE-Cy5 | 1C6/CXCR3 | BD | 551128 | 10 | 25 | Surface | |
| CCR6 | BV605 | 11-A9 | BD Horizon | 562724 | 2.5 | 25 | Surface | |
| KLRG1 (MAFA) | PE-Vio615 | REA261 | Miltenyi | 130-108-395 | 2.5 | 25 | Surface | |
| IL-13 | APC | JES10-5A2 | BD Pharm | 561162 | 0.15 | 30 | Intracellular | |
| Granzyme A | Alx700 | CB9 | Biolegend | 507210 | 0.05 | 30 | Intracellular | |
| TCRva7.2 | APC-Vio770 | REA179 | Miltenyi | 130-100-179 | 5 | 25 | Surface | |
| CD26 | BV711 | M-A261 | BD | 744452 | 1.5 | 25 | Surface | |
| HLA-DR | BUV661 | G46-6 | BD Horizon | 565073 | 0.25 | 25 | Surface | |
| IL-17a | BUV737 | N49-653 | BD Horizon | 624286 | 0.25 | 30 | Intracellular | |
| TCR γδ | BV480 | 11F2 | BD | 746498 | 2.5 | 25 | Surface | |
| CD16 | BV570 | 3G8 | Biolegend | 302036 | 0.5 | 25 | Surface | |
| CD161 | BV650 | DX12 | BD Horizon | 563864 | 2.5 | 25 | Surface | |

^a^ Titer is listed as **μ**L of reagent per 50 **μ**L staining volume.

^b^ All staining conditions were performed at room temperature. Intracellular, post fixation/permeabilization; surface, pre-fixation/permeabilization

**Online Table 3.** Antibodies tested but not used for the final panel.

| **Specificity** | **Fluorochrome** | **Ab clone** | **Vendor** | **Catalog #** | **Titer^1^** | **Reason for exclusion^2^** |
| --- | --- | --- | --- | --- | --- | --- |
| TNF-α | FITC | MAb11 | ebioscience | 11-7349-82 | 0.2 |  |
| CD26 | FITC | M-A261 | BD Pharm | 555436 | 1 |  |
| CD154 (CD40L) | PE-Cy5 | TRAP1 | BD Pharm | 555701 | 2.5 | Significant spread into R660 reducing sensitivity of detection of low expressing Th2 cytokines |
| CD154 (CD40L) | BB630 | TRAP1 | BD Custom | 624294 | - | Poor separation of positive and negative populations |
| CD4 | BB630 | SK3 | BD Custom | 7052772 | 0.05 |  |
| CD4 | BB790 | RPA-T4 | BD Custom | 624296 | 0.01 |  |
| CD8 | BB660 | RPA-T8 | BD Custom | 624295 | 0.02 |  |
| HLA-DR | BB700 | Tu39 | BD Optibuild | 742224 | 0.02 |  |
| PD1 (CD279) | BB700 | EH12.1 | BD Horizon | 566460 | 1.25 |  |
| CD38 | BB790 | HIT2 | BD Horizon | 624296 | 0.1 |  |
| IL-2 | PE | MQ1-17H12 | BD Pharm | 559334 | 2 | IL-22 was a higher priority for the G575 detector |
| TCR γδ | PE-Dazzle 594 | B1 | Biolegend | 331226 | 1 |  |
| CD45RA | PE-Cy5 | HI100 | BD Pharm | 561885 | 2 |  |
| CD45RA | PE-Cy5 | HI100 | BD Pharm | 555490 | 10 |  |
| CXCR3 (CD183) | PE-Cy7 | 1C6/CXCR3 | BD Pharm | 560831 | 2.5 |  |
| CXCR3 (CD183) | PE-Cy5 | 1C6/CXCR3 | BD Pharm | 550967 | - |  |
| CD4 | PE-Cy5.5 | SK3 | ebioscience | 35-0047-42 | 0.5 |  |
| CD8a | PE-Cy7 | RPA-T8 | Biolegend | 3101012 | 0.1 |  |
| Perforin | PE-Cy7 | B-D48 | Biolegend | 353316 | 0.5 |  |
| CD56 | BUV805 | NCAM16.2 | BD Custom | 624287 | 0.25 |  |
| CD56 | BB790 | NCAM16.2 | BD Custom | 624296 | 0.05 |  |
| CD56 | PE-Cy7 | B159 | BD Pharm | 557747 | - | Poor separation of positive and negative populations |
| TCR γδ | PE-Cy7 | B1 | Biolegend | 331222 | 1 |  |
| IL-5 | APC | TRFK5 | BD Pharm | 554396 | 1 | Non-specific binding/background issues |
| CXCR3 (CD183) | APC | 1C6/CXCR3 | BD | 550967 | - | Poor separation of positive and negative populations |
| PD-1 (CD279) | APC-Cy7 | EH12.2H7 | Biolegend | 329922 | 0.05 |  |
| TNF-α | BUV395 | MAb11 | BD Horizon | 563996 | 0.2 |  |
| CCR6 (CD196) | BUV496 | 11-A9 | BD Horizon | 564659 | - | Poor separation of positive and negative populations |
| CD8 | BUV496 | RPA-T8 | BD Horizon | 564804 | 0.5 |  |
| IL-2 | BUV563 | MQ1-17H12 | BD Horizon | 624284 | 0.5 |  |
| CD45RA | BUV563 | HI100 | BD Horizon | 565702 | 1 |  |
| CD4 | BUV661 | SK3 | BD Pharm | 566003 | 0.5 |  |
| CD14 | BUV661 | MPHIP9 | BD Custom | 624285 | 0.5 |  |
| CD45RA | BUV737 | HI100 | BD Horizon | 564442 | 0.15 |  |
| CD3 | BUV805 | SK7 | BD Horizon | 565515 | 0.3 | Down-regulation on stimulated cells |
| CXCR3 (CD183) | BV510 | 1C6/CXCR3 | BD Optibuild | 740183 | - | Poor separation of positive and negative populations |
| CD161 | BV510 | DX12 | BD Horizon | 563212 | - | Poor separation of positive and negative populations |
| KLRG1 (MAFA) | BV605 | 2F1/KLRG1 | Biolegend | 138419 | - | Poor separation of positive and negative populations |
| TNF-α | BV605 | MAb11 | BD Horizon | 563915 | 0.2 |  |
| CCR6 (CD196) | BV650 | 11-A9 | BD Horizon | 563922 | 1.25 |  |
| PD-1 (CD279) | BV711 | EH12.1 | Biolegend | 564017 | 0.3 |  |
| CD56 | BV711 | HCD56 | BD Horizon | 318336 | 0.2 |  |
| CD4 | BV711 | SK3 | BD Horizon | 563028 | 2 |  |
| CXCR3 (CD183) | BV711 | 1C6/CXCR3 | BD Horizon | 563156 | - | Poor separation of positive and negative populations |
| CD4 | BV750 | SK3 | BD | 566355 | 0.03 |  |
| IL-2 | BV750 | MQ1-17H12 | BD Horizon | 566361 | 0.15 |  |

^1^ Titer is listed as **μ**L of reagent per 50 **μ**L staining volume. CD154, IFN-γ, IL-2 and IL-4 reagents were titrated with PHA stimulated PBMC. Staining was done at room temperature for 20 minutes for surface markers or for 30 minutes for intracellular markers.

^2^If no reason is listed, then the reagent was not used due to logistical issues in panel design.

**Developmental Strategy**

As noted in the background, we prioritized markers based on importance in TB immunity. We focused on maintaining sensitivity for the primary functional markers, IFN-γ, IL-2, TNF-α and CD40L, and ,where possible, used the same reagent as in prior HVTN ICS assays. This was possible for IFN-γ conjugated to V450. We had attempted to use CD40L (CD154) conjugated to PE-Cy5 (as used in an older validated panel that worked well, but this diminished the sensitivity to detect the Th2 cytokines in the APC detector (discussed below). IL-2 needed to be moved because acceptable reagents for IL-22 were limited, and PE was a good option since this is a bright fluorochrome and thus optimal for a marker expressed at low levels such as IL-22. Staining for TNF-α is typically very bright and thus we considered it acceptable to move the TNF-α from FITC as in our current ICS assays (1,2) to BV750. The Th2 cytokines were prioritized to APC because the staining for these reagents is not bright and APC conjugates are among the best. Although the intention was to include IL-4, IL-5 and IL-13, IL-5 demonstrated somewhat higher background in the DMSO negative control and was dropped. Further testing of IL-5 indicated that background may not be a problem and this reagent could be added in the future. Beyond these first restrictions, many variations were tested (the final version was version 18). The various versions were first modified based on improving separation for selected reagents. Following this process, spillover spreading issues required further changes in the panel design. These result from measurement error and can be quantified using the spillover spreading error as calculated in FlowJo. Although the FMO control is the optimal method to visualize and detect spillover spreading issues, excessive spillover spreading can often be detected using the fully stained panel. In particular, the BB630 reagent caused many spillover spreading issues into other detectors and this detector (B610) was thus not used, although with further optimization it could likely have been used (Online Figure 1). Because there were limited options for PE-Cy5.5 detected in the G710 detector, this detector was also not used initially (although it was used in our final version (see below). This left 26 detectors (or colors) used in this panel.

|  |
| --- |
| Online Figure 1. Spillover spreading issues caused by BB630 CD4 (in the B610 detector) into multiple other detectors. Each plot shows an FMO for the marker on the Y-axis as conducted for a 27-color version of this assay. |

There was also a spillover spreading issue of the BUV563 (U570 detector) into the PE G575 detector. This was first observed for CD45RA BUV563 into IL-22 PE as shown in Online Figure 2. Ultimately, CD8 was placed on BUV563 with the expectation that a higher gate for PE IL-22 would be needed for CD8+ T cells and thus lowering the sensitivity of detection. This was considered an acceptable compromise since the priority was for sensitive detection of IL-22 from CD4+ T cells.

|  | Online Figure 2. Spreading of CD45RA BUV563 (U570 detector) into IL-22 PE (G575 detector). Although this example was from a fully-stained panel rather than an FMO, the spreading into the G575 was very clear. Note that aggregates in the G575 were gated out and that is the reason for the artificial IL-22 boundary. It is unlikely that gating out the aggregates had any effect of the spreading we observed. |
| --- | --- |

The initial choice of the CD3 reagent conjugated to BUV805 was problematic due to observed lower intensity of the CD3 staining on some of the cytokine-producing cells which we suspected was due to down-regulation of the T cell receptor (TCR) complex from the surface of the cell during the 6-hour stimulation with antigen (Online Figure 3). This issue was evident despite the staining being performed intracellularly which typically allows detection of internalized CD3. We initially suspected that the CD3 down-regulation even with intracellular staining could be due to the antibody clone (SK7) as we had not used that clone in our prior ICS assays. But when an alternate clone (UCHT1) that we had routinely used previously became available as a conjugate to BUV805, the same variation in staining was observed. We therefore suspect that this is a problem with the BUV805 conjugate’s inability to stain intracellularly and thus detect the internalized TCR complex. Subsequently, we chose an alternate fluorochrome conjugate for CD3 (BUV395), and this pattern of varied staining was abolished.

|  |
| --- |
| Online Figure 3. Loss of staining intensity (“down regulation”) of CD3 BUV805 following 6-hour stimulation with CMV pp65 peptide pool. Cells are gated as live lymphocytes, and each graph shows that some of the T cells expressing cytokines or CD40L (CD154) in response to CMV stimulation have lower levels of CD3 staining. |

We were hopeful that version 16 of the panel was our final version. However, there was considerable spreading of the CD154 PE-Cy5 reagent into the Th2 APC-conjugated cytokines as detected in the R660 detector. We thought it would be possible to use an angled gate as shown in Online Figure 4 between the CD154 and the Th2 cytokines, but when we compared the frequency of the CD4+ T cells expressing the Th2 cytokines in the fully-stained panel and the CD154 FMO, the frequency was higher for the FMO, indicating that the angled gate was indeed excluding some of the low level Th2-expressing cells. There was also some concern about the TNF spreading into the IL-17a, but the TNF FMO demonstrated that the ability to detect the IL-17 was not affected, likely because the IL-17a staining was bright enough so that most cells were above the FMO boundary.

|  |
| --- |
|  |
| Online Figure 4. Panel version 16 revealed unacceptable spreading of the CD154 PE-Cy5 into the Th2 cytokine conjugated to APC, but spreading of TNF into IL-17a did not affect the ability to detect the IL-17a. The upper panel shows gated CD4+ T cells stimulated with SEB (top) or unstimulated (bottom). The CD154 FMO on the right detected a higher frequency of Th2+ CD4+ T cells as compared to the fully stained panel on the left. The lower panel shows gated CD4+ T cells stimulated with SEB. The frequency of IL-17+ cells is similar for the TNF FMO and the fully-stained sample. |

Since we did not want to move the Th2 cytokines from APC, we moved CD154 to PE-Cy7. CXCR3 had been on PE-Cy7, and since this marker is much less bright than CD154, we tested it on PE-Cy5. We initially kept CD56 on BB790 (panel version 17) but detected unacceptable spreading of the CD154 PE-Cy7 (G780) into the CD56 (B780). The final version (version 18) moved CD56 to PE-Cy5.5 (G710 detector). This resolved the major spreading issues. Some of the less significant spreading issues were mitigated by the gating scheme as shown in Figure 1. For example, HLA-DR (U660) had considerable spreading into CD3 (U395). Since bright levels of HLA-DR are only expressed on CD3- B cells, we gated CD3 vs. HLA-DR to ensure that none of the bright HLA-DR B cells are included in the CD3+ gate. There was some spreading of TNF (V750) into IL-17a (U730). Fortunately, IL-17a was bright enough to allow for an angled gate as shown in Figure 1. There was some spreading of CD154 (G780) into TCR Vα7.2 (R780), and once again, a slightly angled gate was used.

An additional note related to the panel development concerns the TCR γδ reagent. TCR γδ on PE-Cy7 provided much better separation than the BV510 reagent, but could not be used due to other panel constraints. Also notable is the choice of anti-γδ clone. It has been proposed that many anti-γδ antibodies do not detect all γδ T cell subsets when used in combination with other γδ reagents (3). Wistuba-Hamprecht et al., found the unconjugated form of clone 11F2 was able to detect all Vδ1+ and Vδ2+ T cells. We had initially used the B1 clone, but then changed to the 11F2 clone, using a direct conjugate of this clone.

The gating strategy is described in the legend to Figure 1 but is also shown diagrammatically in Online Figure 5. The goal was to avoid any overlapping subsets. For example, classical CD4+ and CD8+ T cells were gated after gating out γδ T cells, MAIT cells and NK T cells.

|  |
| --- |
| Online Figure 5. Gating strategy used to avoid overlapping subsets. |

As an example of the background (response to the negative control stimulation, or DMSO), Online Figure 6 shows the negative control for the sample shown in Figure 1. The response to SEB as shown in Figure 1 is also included. The gate frequencies were not included in Figure 1 to simplify the figure but are included in Online Figure 6. Also, two of the cytokines, IFN-γ and IL-2 are shown for NKT, γδ, and MAIT cells. Note that although there is no response in the γδ T cells for SEB (as expected), these cells demonstrated a robust response to stimulation with BCG vaccine (data not shown).

|  |
| --- |
| Online Figure 6. Example of the negative control as compared with SEB stimulation for the sample shown in Figure 1. Plots of IFN-γ vs. IL-2 are shown on the right for the less conventional T cells. |

Titration results for reagents in the final panel are shown in Online Figure 7.

|  |
| --- |
| Online Figure 7. Reagent titrations. Typically, ten 2-fold dilutions are tested using cryopreserved PBMC from a healthy volunteer. The titer is shown on the x-axis as volume of antibody added in μl (total staining volume was 50 μl). For cytokine reagents, cells were stimulated for 6 hours with SEB. For most reagents, cells were scatter gated on lymphocytes in the graphs. For CD14, total cells (excluding debris) are shown. For TCR γδ and TCR Vα7.2, cells were counter-stained with CD3 to better visualize the staining. For most reagents, the stain index was calculated and a titer at maximal or near maximal stain index was chosen. The stain index is calculated using the median fluorescence intensity (MFI) of the cells staining positive (pos) for the reagent and those not staining (neg), and the robust standard deviation (rSD) of the negative population using this formula: (MFIpos-MFI-neg)/(rSDneg*2). For those reagents with low frequency of positive cells, the optimal titer was visually chosen taking into account the level of staining among the negative cells. The chosen titer is shown in red; if the titer was between two of the tested titers, the red box is shown at the titer closest to the chosen titer. |

**Assay cross-validation**

The performance of one version of the 26-color ICS assay was compared with the performance of the previous validated 12-color ICS assay (a modified version of OMIP-014) (1) as the reference standard assay. This version of the 26-color assay included CD3 conjugated to BUV805, and to account for the CD3 down regulation, CD3+ gating was performed as shown in Online Figure 3, and a Boolean OR combination of those four gates was used as the CD3+ gate. PBMC samples from 30 individuals from an HIV vaccine trial with known responses to either CMV or Gag were stimulated with CMV pp65 peptide pool, Gag peptide pool, and DMSO and SEB as negative and positive controls, respectively. The primary measurement in this comparison study was the IFN-γ and/or IL-2 production by CD4+ and CD8+ T-cell subsets since that is the primary validated cytokine endpoint for HVTN ICS assays, but other cytokine marginal responses were also compared. Concordance Correlation Coefficients (CCC) were calculated to assess both the linear correlation (precision) and mean shift (accuracy) between the assays, and the Pearson’s correlation coefficient was used to determine the linear correlation of the assays. We consider equivalence established if the 95% lower bound of the CCC and the Pearson’s correlation coefficient are greater than 0.70.

The concordance for CMV-specific IFN-γ and/or IL-2 in both T cell subsets was excellent: CCC (95% lower bound of confidence interval) of 0.940 (0.88) for CD4+ T cells and 0.98 (0.96) for CD8+ T cells. For Gag, the concordance for CD8+ T cells was also excellent with CCC 0.93 (0.82), but was lower for CD4+ T cells with CCC 0.82 (0.64). There was one obvious outlier for Gag-specific CD4+ IFN-γ and/or IL-2 production, and when this outlier was removed the CCC improved markedly to 0.92 (0.84).

**Human Subjects**

PBMC were collected by leukapheresis from individuals enrolled in the Seattle Assay Control (SAC) cohort and cryopreserved. Cryopreserved PBMC from HVTN 505 were used for the cross-validation. The Institutional Review Board of the Fred Hutchinson Cancer Research Center approved these protocols, and local institutional review boards approved the protocol for selected study sites for HVTN 505. Prior to enrollment all volunteers provided written consent after being informed of the nature and possible consequences of the studies.

**Staining Protocol**

Commercial materials:

1. **UViD Fixable Dead Cell Stain Kit (Molecular Probes/Invitrogen; Cat #** L**34962)**

**To prepare UViD stock solution referred to as UViD stock, add 50μl of anhydrous DMSO (supplied with kit) directly to a vial of the UViD stain provided in the kit and resuspend contents of vial. Store frozen aliquots at -20°C.**

1. **BD FACS Lyse Solution, 10× (BD Biosciences; Cat #349202), dilute 1:10 in water.**
2. **BD FACS Perm II, 10× (BD Biosciences; Cat #340973), dilute 1:10 in water.**
3. **Brefeldin A (BFA, Sigma Chemical Co.; Cat #B-7651). Add DMSO (Sigma Chemical Co.; Cat #D-2650) directly to a vial of Brefeldin A (1ml DMSO per 5mg BFA). Dispense aliquots into microcentrifuge tubes and freeze in -20°C freezer.**

**For BFA working solution, dilute stock 1:10 by mixing BFA stock solution with D-PBS. BFA is used at a final concentration of 10 µg/ml in the assay.**

1. **CD28/49d (BD Biosciences; Cat #347690), used at a final concentration of 1µg/ml in the assay.**
2. **Dulbecco’s Phosphate-Buffered Saline solution w/o Ca^++^ and Mg^++^(D-PBS, Gibco BRL Life Technologies; Cat # 14190-144)**
3. **EDTA, 20mM (Fisher Chemicals, Cat #O2793-500)**

**To prepare the EDTA working solution referred to as EDTA solution, weigh 744mg of EDTA on a balance, add to a bottle containing 100mL D-PBS, swirl to dissolve, bring to pH 7.2-7.4 with 1M NaOH.**

1. **Fetal Bovine Serum (FBS), heat inactivated (Gemini Benchmark; Catalog #100-106)**
2. **Golgi Stop containing monensin (BD Cat #554724), concentration not listed by vendor.**
3. **L-glutamine (Gibco BRL Life Technologies; Cat #25030-081)**
4. **Paraformaldehyde, 10% (Electron Microscopy Sciences; Cat #15712-S)**

**To prepare the paraformaldehyde working solution referred to as 1% Paraformaldehyde, dilute the 10% Paraformaldehyde 1:10 in D-PBS.**

1. **Penicillin-Streptomycin (Gibco BRL Life Technologies; Cat #15140-122)**
2. **RPMI 1640 with 25mM HEPES buffer and L-glutamine (Gibco BRL Life Technologies; Cat # 22400-089)**
3. **Staphylococcal enterotoxin B (SEB, Sigma Chemical Co.; Cat #S4881 or Millipore; Cat # 324798-500UG)**

#### To prepare **SEB working solution, add D-PBS directly to a new vial of SEB (2ml D-PBS per 1mg SEB).**

Prepared media:

### Culture media referred to as **R10**

#### Add 55ml of FBS, 5ml of L-glutamine and 5ml of Penicillin-Streptomycin into 500ml of RPMI 1640 with 25mM HEPES buffer and L-glutamine.

### **FACS Wash Buffer**

**Add 10mL heat inactivated FBS to a 500mL bottle of PBS.**

**Procedure:**

1. **Day 1 Sample Thawing**
   1. **On the first day in the afternoon, thaw cryopreserved PBMC, wash and count cells. Resuspend cells in complete media (R10) at** 2**×**10^6^ cells/ml in 50 mL conical tube or culture plate/flask and **place cells in 37ºC 5% CO_2_ incubator overnight.**

### **Day 2 Stimulation**

### **Remove cells from incubator. Wash and count cells and determine cell number and viability.**

- 1. **Resuspend the cells with R10 at 5×10^6^ cells/mL and plate 200 μL/well in a 96 well plate. For the compensation wells, cells from any donor can be used, any remaining cells from different donors can be pooled, or cells from another experiment can be used. Antibody capture beads can also be used.**
  2. **Thaw assay specific peptide pool aliquots by leaving the vials at room temperature up to one hour on the bench top or in the biosafety cabinet.**
  3. **Prepare stimulation cocktails for DMSO (negative control), SEB (or another positive control), and peptide pools. These cocktails are prepared at 10× concentration. Both Brefeldin A and monensin are used. Final concentrations after adding to cells will be: DMSO, same concentration present in peptide pools (typically ≤0.5%); peptide pools, 1 µg/ml for each peptide; SEB, 0.25 µg/ml, CD28/49d, 1 µg/ml; Brefeldin A, 10 µg/ml; monensin, concentration is proprietary and dilution is provided by vendor.**
  4. **Add 20μL of each stimulation cocktail to appropriate wells.**
  5. **Incubate plates undisturbed in an incubator at 37°C, 5% CO_2_ for six hours (+/- 15 min). If other antigens are tested such as recombinant proteins or whole antigens the time of incubation should be increased and optimized. For detection of NK cell functions the stimulation time may have to be increased.**
  6. **Add 20μL of 20mM EDTA to each well and mix well with a multichannel pipette. Wrap plates in foil and store at 4-8°C overnight.**

1. **Day 3 Staining**
   1. **Remove plates from the refrigerator and centrifuge at 750*×g* for 3 min. After centrifugation, flick supernatant from the wells.**
   2. **Prepare the UViD viability dye staining dilution using the appropriate titer for it (staining volume is 50 μL).**
   3. **Add 50μL of the UViD dilution to the appropriate wells including the UViD compensation control and resuspend the cells. Resuspend all other compensation control wells containing cells with 50 μL of PBS.**
   4. **Incubate at room temperature, in the dark for 20 min.**
   5. **Prepare surface antibody cocktail, using the appropriate titer for each particular lot of antibody (staining volume is 50 μL). Before using it, centrifuge the cocktail in a microcentrifuge for 5 min at maximum speed (16,000g for our centrifuge) to get pellet antibody aggregates.**

**Note: Antibody cocktails with brilliant violet (BV) dyes are always prepared just before use, and antibodies conjugated to BV dyes are added last to minimize the possible dye-to-dye interactions.**

- 1. **After the incubation add 150μL PBS to all wells and centrifuge the plates at 750*×g* for 3 min. Flick supernatant from the wells.**
  2. **Add 200μL PBS to all wells. Centrifuge plates at 750*×g* for 3 min and flick supernatant from the wells.**
  3. **Add 50μL antibody solution to the appropriate wells and resuspend the cells. Resuspend compensation wells with 50 μL FACS wash, and then add individual antibodies to the compensation wells.**
  4. **Incubate at room temperature, in the dark for 20 min.**
  5. **Add 150μL FACS Wash Buffer and centrifuge the plates at 750*×g* for 3 min. After centrifugation, flick supernatant from the wells.**
  6. **Add 200μL FACS Wash Buffer and centrifuge plates at 750*×g* for 3 min. Flick supernatant from the wells.**
  7. **Resuspend cells in each well with 100μL of 1× BD FACS Lyse Working Solution.**
  8. **Incubate the plates in the dark at room temperature for 10 min.**
  9. **Add 100μL of FACS Wash Buffer to each well and centrifuge the plates at 750*×g* for 3 min. Flick supernatant from the wells.**
  10. **Resuspend the cells in the wells with 200μL of FACS Perm II Working Solution.**
  11. **Incubate the plates for 10 min in the dark at room temperature.**
  12. **Centrifuge the plates at 750*×g* for 3 min and flick supernatant from the wells.**
  13. **Add 200μL of FACS Wash Buffer to each well and resuspend cells. Centrifuge the plates 750*×g* for 3 min. After centrifugation, flick supernatant from the wells.**
  14. **Add 200μl of FACS Wash Buffer to each well and resuspend cells. Centrifuge the plates at 750*×g* for 3 min. Flick supernatant from the wells.**
  15. **Prepare antibody cocktail for intracellular reagents, using the appropriate titer for each particular lot of antibody. Staining volume is 50µL. Before using it, centrifuge the cocktail in a microcentrifuge for 5 min at maximum speed (16,000g) to pellet antibody aggregates.**

**Note: Antibody cocktails with BV dyes are always prepared just before use, and antibodies conjugated to BV dyes are added last to minimize the possible dye-to-dye interactions.**

- 1. **Add 50μL antibody solution to the appropriate wells and resuspend the cells. Resuspend compensation wells with 50µL FACS wash, and then add individual antibodies to the compensation wells.**
  2. **Incubate at room temperature, in the dark for 30 min.**
  3. **After the completion of incubation, add 150μl of FACS Wash Buffer to all wells. Centrifuge the plates at 750*×g* for 3 min. Flick supernatant from the wells.**
  4. **Add 200μL of FACS Wash Buffer to each well and centrifuge the plates at 750*×g* for 3 min. Flick supernatant from the wells.**
  5. **Resuspend the cells with 150μL of 1% paraformaldehyde working solution.**
  6. **Wrap the plates in foil and place in refrigerator**. Plates can be typically kept in the refrigerator for up to 18 hours, although it has not been tested for this particular panel.
  7. **Acquire the data by flow cytometry directly from the plates using the high throughput sampler (HTS), or manually after transferring cells from the plates to FACS tubes. When using the HTS, be aware of the dead volume (amount left in well after collection), which may depend on your instrument and the plates used, as it may cause incorrect acquisition of sample. We found that the dead volume needed to be at least 50 μL (e.g., the HTS collected 150 μL from the well that had a total volume of 200 μL).**

Literature Cited

1. De Rosa SC, Carter DK, McElrath MJ. OMIP-014: validated multifunctional characterization of antigen-specific human T cells by intracellular cytokine staining. Cytometry A 2012;81:1019-21.

2. Moncunill G, Dobano C, McElrath MJ, De Rosa SC. OMIP-025: evaluation of human T- and NK-cell responses including memory and follicular helper phenotype by intracellular cytokine staining. Cytometry A 2015;87:289-92.

3. Wistuba-Hamprecht K, Pawelec G, Derhovanessian E. OMIP-020: phenotypic characterization of human gammadelta T-cells by multicolor flow cytometry. Cytometry A 2014;85:522-4.
